# Antifungal effects of eugenol on *Candida albicans* adherence to denture polymers

**DOI:** 10.7717/peerj.15750

**Published:** 2023-08-16

**Authors:** Zubaidah Zanul Abidin, Nosizana Mohd Salleh, Wan Harun Himratul-Aznita, Siti Fauzza Ahmad, Ghee Seong Lim, Noorhayati Raja Mohd, Nabihah Dziaruddin

**Affiliations:** 1 Department of Restorative Dentistry, Universiti Malaya, Kuala Lumpur, Kuala Lumpur, Malaysia; 2 Department of Oral and Craniofacial Sciences, Universiti Malaya, Kuala Lumpur, Kuala Lumpur, Malaysia; 3 Department of Paediatric Dentistry & Orthodontics, Universiti Malaya, Kuala Lumpur, Kuala Lumpur, Malaysia

**Keywords:** *Candida albicans*, Denture base polymers, eugenol, Denture cleansers, Fungal adherence

## Abstract

**Background:**

The study’s objective is to assess the adherence of *C. albicans* in different types of denture polymers and the effectiveness of eugenol and commercialized denture cleansers in the removal of *C. albicans*. Three types of denture base polymers (Lucitone^®^ 199 (High-Impact PMMA), Impact^®^ (conventional PMMA) and Eclipse^®^ (UDMA)) and two hard denture reline materials (Kooliner^®^ and Tokuyama^®^ Rebase II Fast) were used in this study.

**Methods:**

Three hundred samples were prepared (6 × 2 mm disc shape) and divided into five groups of denture polymers (*n* = 60) and further subjected into five treatment groups (Polident^®^, Steradent, distilled water, eugenol 5-minutes, and eugenol 10-min). Three samples were extracted from each treatment group for baseline data (*n* = 12). Baseline data were used to calculate the initial number of *C. albicans* adherence. A 0.5 ml immersion solution from each specimen was cultured on YPD agar and incubated for 48 h at 37 °C. Visible colonies were counted using a colony counter machine (ROCKER Galaxy 230).

**Results:**

The result showed that the denture base polymer significantly affected the initial adherence (*p* = 0.007). The removal of *C. albicans* was also considerably affected by the denture base polymers and denture cleansers (*p* < 0.05). Lucitone^®^, Tokuyama^®^, and Kooliner^®^ denture base polymers immersed for 3 min in eugenol showed the best results of removal.

**Discussion:**

This study’s overall results showed that all denture polymers used as denture bases had an effect on *C. albicans* initial adherence and removal from the denture base, and eugenol is comparable to commercialised denture cleansers in reducing the number of attached *C. albicans* on denture base polymers.

## Introduction

According to Zain et al. (1997) denture stomatitis is among the most common oral mucosa lesions diagnosed in Malaysia, with a high prevalence of approximately 33.5%, while Al-Maweri et al. (2013) reported a lower prevalence of denture stomatitis in patients attending Hospital Universiti Sains Malaysia. These different may be due to the latter study used a convenience sample and the former study samples were randomly sampled from national population.

Denture stomatitis is a lesion usually seen on the maxilla and mandibular denture-bearing area, and as the name suggested, it is related to wearing dentures. However, it is more common on the hard palate of the maxillary denture compared to the mandibular region due to more area of contact between the maxillary denture and oral tissues. The causes of this lesion are mainly due to poor oral hygiene of denture wearers, continuous or prolonged wearing of the denture, particularly overnight, wearing poorly constructed dentures, pharmacological reaction (allergic reaction), and *C. albicans* infection in combination with all the factors mentioned above.

*C. albicans* is the most common species found as commensal members in the microflora of a healthy human. According to Scully & Felix (2005), *C. albicans* is the most common species isolated from denture stomatitis cases. The presence of saliva and its flow help in the self-cleansing effect on the oral mucosa. However, the presence of pellicle promotes the adherence of *Candida* spp. to the denture base polymer. In patients with xerostomia, artificial saliva is used to lubricate the oral environment, to keep the mouth moist, and to improve self-cleansing of the oral cavity. A study by Hahnel et al. (2010) reported that saliva substitute films influence the initial adherence of *C. albicans* and the artificial saliva with lysozyme and lactoferrin may be effective in preventing *C. albicans* from adhering to oral surfaces. Hence, artificial saliva with these substitutes could serve as a first step in a therapy plan targeting the oral microbial including *C. albicans*.

A study by De Freitas Fernandes et al. (2011), reported that polymethyl methacrylate resin surface of denture polymer may promote on microbial colonization. Many studies had shown that surface roughness of denture base material or denture liner does have an impact on microbial adherence (Meirowitz et al., 2021; Rapone et al., 2022; Di Fiore et al., 2022). A study by Verran & Maryan (1997) has found less adherence of *Candida* spp on rough surface of denture base than the smoother surface. However, Moura et al. (2006) had reported that surface roughness and polymerization method have no impact on *Candida* adherence, but the presence of saliva does have an impact on *Candida* adherence.

Many over-the-counter denture cleansers have been developed recently with more added functions such as effervescent effect (mechanical plaque removal by bubbling effect) or fluoride additive (prevention of dental caries especially in partial dentate patients) and rapid action to clean the denture. Ramage et al. (2019) compared two denture cleansing methods, using a denture cleanser alone and brushing with a dentifrice, and found that using a denture cleanser daily is more effective than brushing with dentifrice to inhibit the growth of *C. albicans* on denture surface.

In the last century, researchers found that eugenol (4-allyl-2-methoxy phenol), the main compound of clove (*Syzygium aromaticum*), has antibacterial and antifungal properties (Kim, Marshall & Wei, 1995; Ouattara et al., 1997). eugenol is a polyphenol component that can be found in a variety of plant sources, including clove oil which has medicinal and nutritional benefits (Ohkubo & Shibata, 1997; Rana, Rana & Rajak, 2011). It is also can be obtained in other sources such as clove or African Basil plant (*Ocimum gratissium*), holy basil plants (*Ocimum tenuiflorum, Ocimum sanctum*), Golden Shower flowering plant (*Cassia fistula*), lanoline bush plant (*Zieria smitii*), fairy fans wildflower plant (*Clarkia breweri*), Billygoat weed plant (*Ageratum Conyzoides*) and also in whole tobacco plant (Reddy et al., 2007).

The mechanism by which eugenol triggers mortality in *Candida* spp are uncertain and not fully understood. The chemical compound in eugenol generated significant morphological alterations in *Candida* spp cells and cytoplasmic component leakage, indicating an effect on the cell membrane (Nakamura et al., 2004; Chami et al., 2005). Several authors have demonstrated that fungicidal concentrations of eugenol against *C. albicans* cause a significant decrease in cell ergosterol content which may interfere with H+-ATPase activity (Pinto et al., 2009; Ahmad et al., 2010a; Ahmad et al., 2010b; Khan, Ahmad & Cameotra, 2013). It is possible that the primary purpose of eugenol to destroy the permeability of cell membranes, leading them to degrade or dissolve. Additionally, previous studies have demonstrated that eugenol can substantially increase the permeability of phospholipid bilayers in cell membranes (Woranuch & Yoksan, 2013; Wang et al., 2010). Braga et al. (2007) also found that eugenol could alter the *C. albicans* cell envelope, thus reducing the ability of *C. albicans* to colonise host tissue; the cell envelope referred to combination layer of the plasma membrane, the periplasmic space of the cells, the cell walls of also the fibrous layer on the outer region of the *C. albicans* wall. The eugenol resulted in a significant alteration in the morphology of the envelope of *C. albicans*. According to that study, the mechanism is still unclear but may be due to the present of lipophilic and carvacrol compounds founds in eugenol that change the fluidity and permeability of cell membranes by penetrating between the fatty acyl chains that make up the lipid bilayers.

Because of its diverse range of biological and functional properties, eugenol may be a topic of considerable interest. Therefore, the present study aims to investigate the effectiveness of eugenol and commercialised denture cleansers in reducing the initial adherence of *C. albicans* to denture polymers.

## Materials & Methods

### Microbiological preparation

*C. albicans* (ATCC 14053) (The American Type Culture Collection (ATCC), USA) was used in adherence assay in this study. The strain was rehydrated in sterile distilled water and inoculated onto Yeast Peptone Dextrose (YPD) agar media (BD Difco, Franklin Lakes, NJ, USA). This was followed by incubation at 37 °C for 24 h, and then the colonies were subcultured on fresh YPD agar slants and stored at 4 °C. Stocks for long-term storage were also prepared in 20% glycerol and kept at −70 °C.

### Denture based polymers

Three types of denture base polymers (Lucitone^®^ 199 (High-Impact heat-polymerised PMMA), Impact^®^ (conventional heat-polymerized PMMA) and Eclipse (UDMA-based light-cured resin)) (Table 1) and two types of hard denture reline polymers (Kooliner^®^ and Tokuyama^®^ Rebase II Fast) (Table 2) were used in this study. Sixty-five denture base samples, discs of size 6.0 mm diameter × 2.0 mm height, were fabricated from each denture base (*n* = 325). Samples were fabricated according to the manufacturer’s instructions and polished for further use in the experiment. For the polishing procedure, the discs were trimmed with grind and polishing machine (Buehler Beta, Lake Bluff, IL, USA) using 240 grit silicon carbide (3 min for both sides at 60 rpm) followed with 1000 grit silicon carbide (5 min for both sides at 300 rpm) until reaches to a final thickness of 1 ± 0.2 mm. Digital electronic caliper (Mitotuyo Digimatic Vernier Caliper, Mitotuyo Corp, Kawasaki, Japan) was used to control thickness for each specimen. Grinding and polishing procedure were performed by the same operator with constant motion and light minimal pressure.

**Table 1:** Denture base polymers.

Material type	Product Name	Main composition	Manufacturer	Batch number
High-Impact heat-polymerizing Polymethyl methacrylate	Lucitone^®^ 199	Powder: Polymethylmethacrylate Liquid: Methylmethacrylate, ethylene glycol dimethacrylate	Dentsply, International Inc. York, PA, USA.	Powder: Lot 120220 Liquid: Lot 111026
Conventional heat-polymerizing Polymethyl methacrylate (PMMA)	Impact^®^ Acrylic Denture Base	Powder: Polymethylmethacrylate Liquid: MMA, ethylene glycol dimethacrylate	Dental export of London, London, England.	Powder: Lot 11NOV147 Liquid: Lot 22745
Light Activated Urethane Dymethacrylate (UDMA)	Eclipse^®^ Base Plate	Single paste component Urethane dimethacrylate, Stearyl Acrylate	Dentsply, International Inc. York, USA.	Lot 030909

**Table 2:** Hard denture relines.

Polymer	Product name	Main composition	Manufacturer	Batch number
Hard denture reline	Kooliner^®^	Powder: Polyethyl methacrylate, Benzoyl peroxide, Silica, Crystalline-Quartz Liquid: Isobutyl methacrylate, 2,4-Dihydroxy benzophenone	GC Dental Product	Lot 1201079
Hard denture reline	Tokuyama^®^ Rebase II	Powder—Polyethyl methacrylate, Benzoyl peroxide Liquid-2-(Acetoacetoxy) ethyl methacrylate and 1,9-nonanediol Dimethacrylate Tokuso Resin Hardener II—sodium sulfite and sodium bicarbonate	Tokuyama^®^ Dental America Inc.	Lot 139E62

### Sample groups

Next, denture base samples were randomly divided into five groups (*n* = 13 per group). All samples were soaked in chlorhexidine 0.12% for one hour, and were washed thoroughly with distilled water, and subjected to sonication methods for 10 min and were repeated ten times consecutively (Table 3). One specimen from each denture base group was taken aside and not subjected to any adherence assay and used as a negative control. The remaining twelve samples from each denture base group were subjected to the adherence assay for 3 h at 37 °C with *C. albicans* suspension prepared at an optical density (OD550nm) of 0.144, which is equivalent to 10^6^ cells/ml. Before that, the samples were soaked in artificial saliva for 30 min (Table 3). Three samples were extracted from each treatment group for baseline data (*n* = 15) and labelled as E1. Baseline data were used to calculate the initial number of *C. albicans* adherence. The denture base polymers were then soaked in five types of denture cleansers Polident^®^, Steradent, sterile distilled water, eugenol 3 min, and eugenol 10 min and labelled as E2 (Table 4). 0.5ml of the immersion solution from each specimen of E1 and E2 groups were then cultured on YPD agar and incubated for 48 h at 37 °C.

**Table 3:** Other solutions.

Solution	Product Name	Main composition	Manufacturer	Batch number
Artificial saliva	Kin Hydrat Spray	Xylitol, Potassium Chloride, Sodium Chloride, Calcium Chloride, Potassium Thiocyanate, Sodium Saccharin	LABORATORIOS KIN S.A, Barcelona, Spain	Lot 12C01
YPD Broth	Difco YPD Broth	Yeast extract, Peptone, Dextrose	Difco Laboratories	Lot 2100348
Chlorhexidine 0.12%	Oradex antibacterial mouthwash	Chlorhexidine Gluconate	Fortune Laboratories Sdn. Bhd	Lot 1212057B

**Table 4:** Denture cleansers.

Product name	Main Composition	Manufacturer	Batch Number
Polident^®^	Citric Acid, Sodium Carbonate, Potassium Peroxymonosulfate, Sodium Perborate Monohydrate	Glaxo Smith Kline	Lot FD999101
Steradent	Potassium Peroxymonosulfate Sulfate, Sodium Carbonate, Citric Acid, Sodium Carbonate Peroxyhydrate, Sodium Sulfate, Sulfamic Acid, Malic Acid.	Reckitt Benckiser	Lot 0308943
eugenol	2-methoxy-4-(2-propenyl)phenol* 4-Allyl-2-methoxyphenol	Fluka Analytical	Lot BCBD3793
Sterile Distilled Water (dH_2_O)	Hydrogen, Oxygen		

### Adherence analysis

Visible colonies were counted using a colony counter machine (ROCKER Galaxy 230) and represented as colony-forming units (CFU).

### Data analysis

Data collected were entered and analysed in SPSS version 22. The assumption for normality was not met; therefore, non-parametric analysis was used. The Kruskal-Wallis test was used to analyse the initial adherence of *C. albicans* to the denture polymers. To analyse eugenol’s effectiveness and commercialised denture cleanser on the removal of *C. albicans*, the Mann–Whitney U test was used with Bonferroni correction for multiple comparisons. A statistically significant level was set to a *p* value of 0.05.

## Results

### Initial adherence of *C. albicans* on different types of denture polymers

The amount of initial adherence of *C. albicans* on different types of denture polymers are shown in Table 5.

**Table 5:** Initial adherence of *Candida albicans* on the different type of denture polymers.

Denture polymer	Initial Adherence (CFU/ml)	Kruskal-Wallis		
	Median ± IQR (10^2^)	**X** ^2^	**Df**	***p*-value**
Lucitone^®^	24 ± 4^a,b,e^	47.70	4	0 .000
Impact^®^	20 ± 3^a,c^			
Eclipse^®^	22 ± 9^e^			
Kooliner^®^	21 ± 3^b,d^			
Tokuyama^®^	24 ± 8^c,d^			

**Notes.**

Significant at *p* < 0.05.

a,b shows significant different differences between groups (post hoc analysis using Mann–Whitney U with Bonferroni correction).

A Kruskal-Wallis H test showed that there was a statistically significant difference in the initial adherence of *C. albicans* among different denture polymers, *X*^2^(4) = 47.70, *p* < 0.001. The initial adherence of *C. albicans* to denture polymers ranges from 20 ± 3 CFU/ml (Impact) to 24 ± 8 CFU/ml (Tokuyama) with a mean rank initial adherence of 161.60 for Lucitone 199^®^, 131.00 for Tokuyama, 104.00 for Eclipse, 89.60 for Kooliner and 78.80 for Impact.

Post hoc analysis showed that the initial adherence of *C. albicans* on Impact^®^ (20 ± 3 CFU/ml) and Kooliner^®^ (21 ± 3 CFU/ml) were significantly lower than Lucitone 199^®^ (*p* = 0.007). However, no statistically significant difference was observed in the initial adherence of *C. albicans* on the Lucitone 199^®^, Eclipse and Tokuyama^®^ denture polymers (*p* > 0.05).

### Effectiveness of eugenol and commercialised denture cleansers in removing *C. albicans* from denture base polymers

The effectiveness of eugenol and denture cleansers in removing *Candida* was measured by the amount of *C. albicans* removed from the denture base polymers in CFU/ml. The amount of *C. albicans* removed from the denture polymers varies among the groups and ranges from 2,740 ± 30 CFU/ml to −53 ± 685 CFU/ml upon immersion in the denture cleansers, as shown in Table 6. The negative value shown in distilled water would suggest the growth of *Candida*. The removal of *Candida* is significantly affected by the denture base and denture cleanser (*p* < 0.001).

**Table 6:** The effectiveness of eugenol and commercialised denture cleanser on the removal of *Candida albicans* on denture base polymers.

Denture base	Denture Cleanser	Colonies removed (CFU/ml)	Kruskal-Wallis		
		Median ± IQR	X^2^	df	*p*-value
Impact^®^	Polident^®^	2140 ± 55^a,b^	40.05	4	<0.001
	Steradent	1863 ± 30^b,c^			
	Eug-3	1983 ± 10^d^			
	Eug-10	2010 ± 5^c,e^			
	dH_2_O	67 ± 325^a,d,e^			
					
Lucitone^®^	Polident^®^	2267 ± 15^f,g^	42.67	4	<0.001
	Steradent	2140 ± 20^h,i^			
	Eug-3	2650 ± 10^g,h,j^			
	Eug-10	2523 ± 0^i,k^			
	dH_2_O	260 ± 365^f,j,k^			
					
Eclipse	Polident^®^	2740 ± 30^l,m,n^	42.56	4	<0.0001
	Steradent	1700 ± 50^n,o^			
	Eug-3	2163 ± 10^p,q^			
	Eug-10	1590 ± 5^m,q^			
	dH_2_O	67 ± 730^l,o,p^			
					
Tokuyama^®^	Polident^®^	1837 ± 75^r^	40.30	4	<0.001
	Steradent	1803 ± 140^s^			
	Eug-3	2733 ± 10^r,s,u^			
	Eug-10	2600 ± 10^t^			
	dH_2_O	410 ± 425^t,u^			
					
Kooliner^®^	Polident^®^	1960 ± 5^v^	40.12	4	<0.001
	Steradent	2060 ± 10^w^			
	Eug-3	2313 ± 10^x,y^			
	Eug-10	1823 ± 10^y^			
	dH_2_O	−53 ± 685^v,w,x^			

**Notes.**

Eug-3: 3 min immersion in eugenol.

Eug-10: 10 min immersion in eugenol.

Significant at *p* < 0.05. Superscript letters show significant different between groups (post hoc analysis using Mann–Whitney *U* with Bonferroni correction).

Impact^®^ denture base polymer

For Impact^®^ denture base, immersion in all denture cleanser solutions significantly removed *C. albicans* colonies by at least 28 folds compared to distilled water (67 ± 325 CFU/ml) (*p* < 0.05). There is no significant difference between three- and ten-minute immersion in eugenol with pairwise comparison (*p* > 0.05) and both did not differ significantly with other cleansers except between ten minutes immersion in eugenol (2010 ± 5 CFU/ml) and Steradent (1863 ± 30 CFU/ml).

Lucitone 199^®^ denture base polymer

Immersion of Lucitone 199^®^ denture base in three- and ten-minute immersion in eugenol significantly removed more *C. albicans* colonies (2650 ± 10 CFU/ml and 2523 ± 0 CFU/ml respectively) compared to other denture cleansers and they did not differ significantly. However, immersion in eugenol for three minutes significantly removed *C. albicans* compared to Polident^®^ and Steradent, and immersion in ten minutes eugenol only differ significantly from Steradent denture cleanser.

Eclipse denture base polymer

Three minutes of immersion in eugenol removed more *C. albicans* (2,163 ± 10 CFU/ml) compared to ten minutes of immersion (1,590 ± 5 CFU/ml) and the difference was significant (*p* < 0.05). Immersion in ten minutes of eugenol also differs significantly with Polident^®^ (2,740 ± 30 CFU/ml).

Tokuyama denture base relining material

No significant difference was found between three- and ten-minute immersion in eugenol (2,733 ± 10 CFU/ml and 2,600 ± 10 CFU/ml respectively). However, three minutes of immersion in eugenol differ significantly from other cleansers (Steradent (1,803 ± 140 CFU/ml) and Polident (1,837 ± 75 CFU/ml)) (*p* < 0.05). The colonies of *C. albicans* removed from the Tokuyama^®^ denture base after immersion in distilled water (44 ± 685 CFU/ml) are four folds less compared to after immersion in other denture cleansers (*p* < 0.05).

Kooliner denture base relining material

For Kooliner^®^ denture base, the highest number of *C. albicans* colonies removed was after three minutes of immersion in eugenol (2,313 ± 10 CFU/ml), which was significant compared to the ten minutes of immersion in eugenol (1,823 ± 10 CFU/ml), and both differ significantly. Both three- and ten-minute immersion did not differ significantly from other denture commercial denture cleansers.

**Figure 1:**
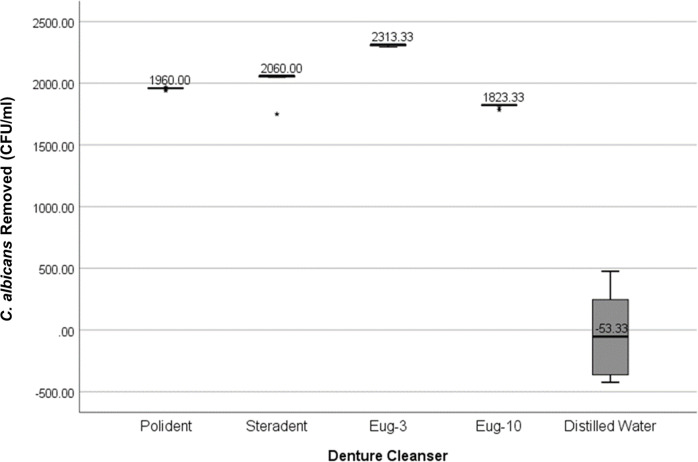
Graph of *C. albicans* removed (CFU/ml) by each denture cleanser. The amount of *C. albicans* removed after immersion in denture cleansers from all types of denture base materials.

**Figure 2:**
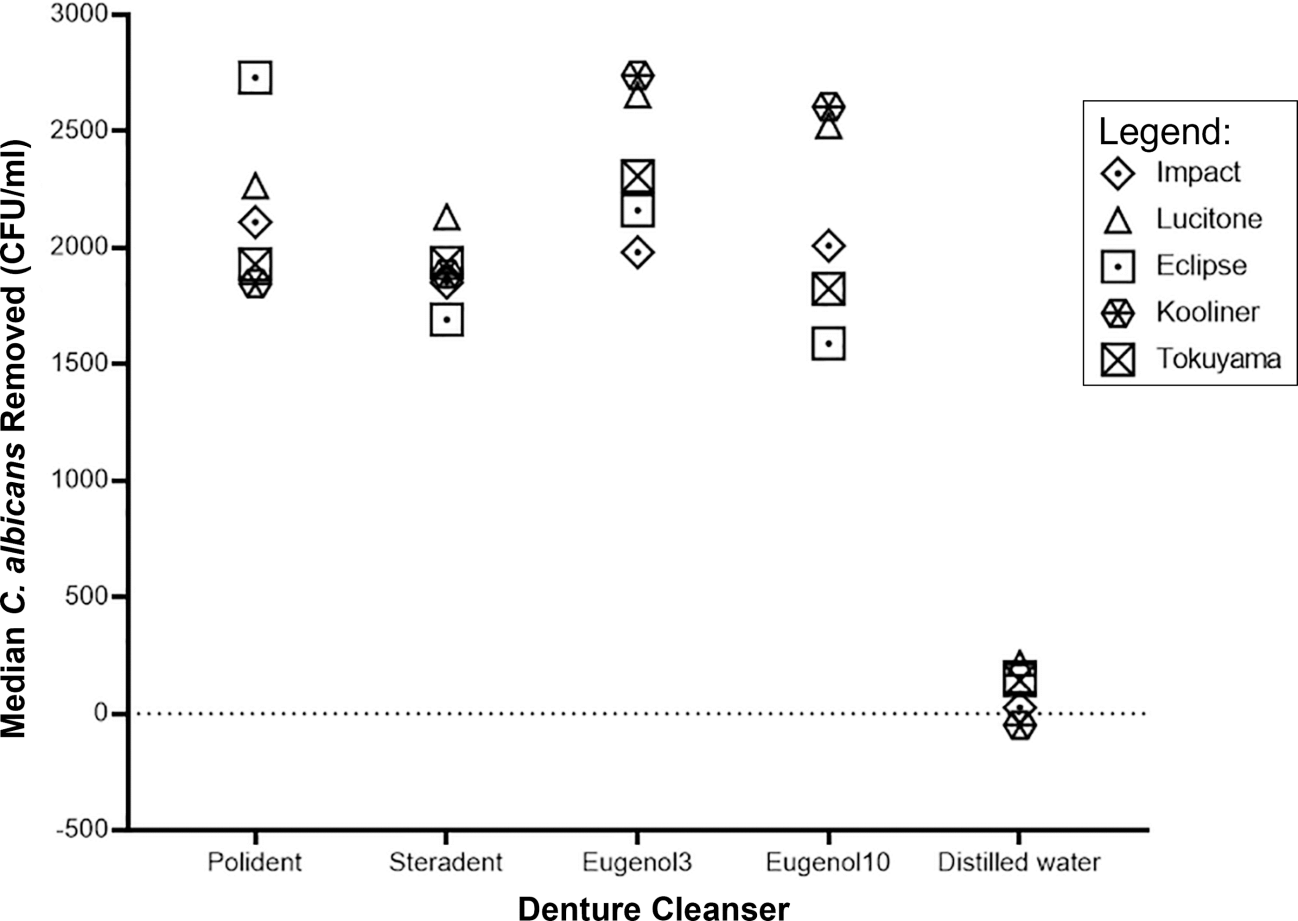
Graph of median *C. albicans* removed (CFU/ml) by denture cleanser. The median of *C. albicans* removed after immersion in denture cleansers varies in denture bases.

The effect of denture cleansers varies with different types of denture base polymers. In general, immersed in distilled water removed the least number of *C. albicans* whilst immersed in eugenol solution for three minutes yields the greatest number of *C. albicans* removed from all the denture bases (Figs. 1 and 2).

## Discussion

Microbial adherence to denture polymers surfaces can be measured by using several different quantitative methods such as colony-forming unit (CFU) assay, scanning electron microscopy (SEM), fluorescence microscopy, crystal violet assay or optical density (OD) (Cheng et al., 2019). The colony-forming unit (CFU) assay used in this study provided a simple and reproducible method commonly used for measuring the initial adherence between *Candida* and denture polymers (Sieuwerts et al., 2008).

The adherence of *Candida* to denture base can be affected by various factors, such as the experiment environment (*in-vivo* or in-vitro) and the physical and mechanical properties of the denture materials to name a few (Gacon, Loster & Wieczorek, 2019; Sampaio-Maia et al., 2012; Radford et al., 1998). In the present study, the experiment was conducted *in-vitro* with more precise control of experimental conditions, but the initial adherence may depend mainly on the denture polymers itself. Yildirim et al. (2005) reported that in the adherence of *Candida* to solid surfaces, both hydrophobic and electrostatic interactions are involved depending on the physiochemical properties of the surfaces of both solids and *Candida*.

Three different denture base materials used in this study were among the commonly used materials for the fabrication of denture base. The light-activated urethane dimethacrylate (UDMA) (Eclipse^®^) was used with a different curing mode. A survey by Hashem et al. (2014) reported that UDMA denture base polymer has better mechanical and physical properties in terms of surface hardness and flexural strength than PMMA. This material is a timesaving since it eliminates the investing, de-waxing, and packing in flask stages as in the conventional method. Besides that, it has a shorter curing time compared to PMMA.

### The initial adherence of *C. albicans* on different types of denture polymers

Our study showed that denture base polymer type significantly affected the initial adherence of *C. albicans*; the initial *C. albicans* adherence to denture polymers ranged from 20 ± 3 CFU/ml (Impact) to 24 ± 8 CFU/ml (Tokuyama^®^). In contrast to our findings, Nevzatoğlu et al. (2007) found that the *C. albicans* adherence was higher for cold-polymerized resins than heat-polymerized ones, regardless of the surface finishes. These discrepancies in the results may be partly attributed to the differences in the physicochemical properties of the denture polymers and *Candida* species used. The chemical composition of the denture polymers surface may also influence *C. albicans* adhesion much more than the average surface roughness (Yildirim et al., 2005). This result contradicts the previous study by Devarhubli, Subbarao & Patil (2012), who reported a lower adhesion of *Candida* in Lucitone 199^®^ heat-cured denture base material.

The lowest value of adhesion between the two reline materials was found with Kooliner^®^ (21 ± 3 CFU/ml), while Tokuyama^®^ Rebase II Fast recorded a value of 24 ± 8 CFU/ml. The result coincides with the previous study for the initial adherence study, although artificial saliva was used in the present study to replace the human saliva sample. Hahnel et al. (2010) and Silva et al. (2012) reported that artificial saliva did promote adherence of *C. albicans* to denture base material. Artificial saliva was used because whole human saliva might cause a particular problem due to its complex biochemical and physical-chemical properties; its inherent variability and instability would also be posed as a challenge that will affect the study (Schipper, Silletti & Vingerhoeds, 2007). Therefore, human saliva could be inappropriate for a standardised *in vitro* study; rather artificial saliva may be essential for a well-justified and controlled experiment (Pytko-Polonczyk et al., 2017).

### The effectiveness of eugenol and commercialised denture cleansers in removing *C. albicans* from denture base polymers

Four different denture cleanser solutions, namely Polident^®^, Steradent, eugenol (3-minutes and 10-minutes immersion time) and distilled water (control group), were used to determine the effectiveness in removing *C. albicans* after initial adherence to denture polymers. The concentration of 2% eugenol was chosen in the study as it was reported that 2% clove oil could inhibit the growth of some mycotoxigenic molds and yeasts (Matan et al., 2006; Mandras et al., 2016; Marchese et al., 2017).

Distilled water was used in this study as a control group and as predicted immersion in the distilled water showed lowest removal value of *C. albicans* compared to the study group for all denture base polymers. This exhibited that distilled water alone has no capacity to eliminate the *C. albicans* from the denture polymer surfaces.

The data described from this study indicates that all four cleansers effectively removed the *C. albicans* greater than 70% from the denture polymers surfaces regardless the immersion time. This result was agreeable with the previous study by De Freitas Fernandes et al. (2011), who reported that a denture cleanser could reduce *Candida* spp. cells count on denture polymers. Ghazal et al. (2019) also stated that denture cleanser could effectively detach the adhered *C. albicans* from denture base resin. The active ingredients in Polident^®^ and Steradent^®^ might help to eliminate some of the *Candida* colonies (Han, Liu & Cai, 2020). Polident^®^ contains sodium perborate, a hydrogen peroxide source. When these tablets are dissolved in water, sodium perborate forms an alkaline peroxide solution. This solution releases oxygen, which loosens debris or adherence of microorganisms by mechanical means.

Eugenol was significantly more effective than other denture cleansers in removing *C. albicans* from Lucitone^®^ and Tokuyama^®^ denture base polymers, even after 10 min immersion. This was supported in the previous study by Fontenelle et al. (2011), who reported that eugenol was significantly active against *Candida* spp. Being an antifungal agent, eugenol could inhibit the growth of *C. albicans* (Silva et al., 2017) and showed good potential to inhibit spoilage fungi, yeast, and bacteria (El-Saber Batiha et al., 2020). eugenol was also found to modify the cell membrane’s morphology of *C. albicans*, thus reducing its colonization on host tissues (Latifah-Munirah, Himratul-Aznita & Mohd Zain, 2015; Rajkowska, Nowicka-Krawczyk & Kunicka-Styczyńska, 2019). Although, the longer immersion time may lead to deleterious effects on the denture base polymers or not, could not be concluded without further study. This study demonstrated a significant greater *C. albicans* removal value following a 3-minute immersion compared to 10-minute immersion in eugenol (*p* < 0.001), suggesting 3 min immersion time is acceptable for eugenol. Lucitone^®^, Tokuyama^®^ and Kooliner^®^ denture base polymers immersed for 3 min in eugenol showed the best results of removal.

## Conclusions

This study demonstrated that *C. albicans* could adhere to all denture polymers even with artificial saliva. Different types of denture polymer polymerising systems allowed the different number of *C. albicans* in the initial adherence. All denture cleansers in the present study were suitable for all denture polymers in reducing the number of attached *C. albicans*. It is safe to say that eugenol was as effective as commercialised denture cleansers in reducing the number of attached *C. albicans* on denture polymers. This is because eugenol is a potent antifungal, especially to *C. albicans* besides, as an effective pain reliever. However, there are a few limitations with utilizing eugenol as the base compound for the potential denture cleanser, such as allergic reaction, and an unpleasant taste of spiciness, to name a few. Further studies could be recommended to investigate the addition of some materials to neutralize the effect of the eugenol. Lower percentage and less immersion time could also be investigated to overcome these limitations. This study design did not simulate the clinical situation ideally, as artificial saliva was used to replace human saliva to form the pellicle for attachment of *C. albicans*. Further randomized clinical trials are recommended to assess the effect of eugenol as denture cleanser inside the oral environment.

Furthermore, the surfaces of the denture polymer used in this study have been polished to a smooth finishing, standardising the surfaces. In contrast, in clinical situations, the surface of the intaglio surface was rougher, which might promote the adherence of *C. albicans*. Therefore, it is necessary to conduct further studies to simulate the actual clinical situation.

##  Supplemental Information

10.7717/peerj.15750/supp-1 File S1 Raw data

## Additional Information and Declarations

### Data Availability

The following information was supplied regarding data availability: The raw measurements are available as Supplemental File.
